# About Merging Threshold and Critical Flux Concepts into a Single One: The Boundary Flux

**DOI:** 10.1155/2014/656101

**Published:** 2014-01-29

**Authors:** Marco Stoller, Javier M. Ochando-Pulido

**Affiliations:** ^1^Department of Chemical Materials Environmental Engineering, University of Rome “La Sapienza”, Via Eudossiana 18, 00184 Rome, Italy; ^2^Chemical Engineering Department, University of Granada, 18071 Granada, Spain

## Abstract

In the last decades much effort was put in understanding fouling phenomena on membranes. One successful approach to describe fouling issues on membranes is the critical flux theory. The possibility to measure a maximum value of the permeate flux for a given system without incurring in fouling issues was a breakthrough in membrane process design. However, in many cases critical fluxes were found to be very low, lower than the economic feasibility of the process. The knowledge of the critical flux value must be therefore considered as a good starting point for process design. In the last years, a new concept was introduced, the threshold flux, which defines the maximum permeate flow rate characterized by a low constant fouling rate regime. This concept, more than the critical flux, is a new practical tool for membrane process designers. In this paper a brief review on critical and threshold flux will be reported and analyzed. And since the concepts share many common aspects, merged into a new concept, called the boundary flux, the validation will occur by the analysis of previously collected data by the authors, during the treatment of olive vegetation wastewater by ultrafiltration and nanofiltration membranes.

## 1. Introduction

Critical and threshold flux theories were quickly applied by researchers and membrane process designers in order to inhibit membrane fouling in many systems. As an illustration of popularity of the use of these concepts, more than 6400 papers were published in international scientific journals in the last 5 years [1].

Membrane fouling still remains nowadays one of the main challenges of the broad applied membrane technology, especially in liquid-liquid separation processes [2]. Membrane fouling may lead to dramatically shorten the life time of membrane modules. For this reason, engineers design membrane processes with an excessive oversized capacity, up to 35%, increasing both investment and operating costs [3]. In other words, the lack of knowledge and control of membrane fouling are an additional cost for the industry which should be minimized to permit successful competiveness of the technology. This applies especially on wastewater purification processes [4].

Field et al. introduced the concept of critical flux for microfiltration, stating that there is a permeate flux below which fouling is not promptly observed [5]. Afterwards, it was possible to identify critical flux values on ultrafiltration (“UF”) and nanofiltration (“NF”) membranes systems too [6]. Nowadays, the critical flux concept is well accepted by both scientists and engineers as a powerful membrane process optimization tool [7].

The main drawback of this concept is that the determination of critical flux values can not be theoretically predicted, but only experimentally measured by time consuming experiments. Moreover, different critical flux values can be measured on the same system, depending on various factors, such as hydrodynamics, temperature, feed stream composition, and membrane surface characteristics [8–10]. Feed stream composition is the main responsible of variable critical flux values in case of agricultural wastewater stream treatment by membranes, since the entering feedstock quality is not constant during time. Moreover, the use of batch membrane processes in order to limit the amount of required membrane area and thus saving investment costs leads to sensible feedstock changes during operation. As a consequence, critical flux values never remain constant, which represent a major difficulty in fine-tuning optimal operating conditions.

In case of many real wastewater streams, Le-Clech et al. noticed that operations below the critical flux may not be sufficient in order to have zero fouling rates. Therefore, it appears that membrane systems treating real wastewater streams do not exhibit a critical flux in a strict way [11]. To overcome this limitation in the definition of critical flux, in a recent paper, Field and Pearce introduced for the first time the concept of threshold flux [12]. Summarizing briefly the concept, the threshold flux is the flux that divides a low fouling region, characterized by a nearly constant rate of fouling, from a high fouling region, where flux dependant high fouling rates can be observed.

In the past years, before the concept of the threshold flux was introduced, the author published many papers on olive wash wastewater (“OWW”) purification by membranes, mainly ultrafiltration and nanofiltration, always determining critical fluxes [13–15]. The critical flux concept appears to be valid, and it was possible to achieve optimization of the membrane system by using the measured data. In the studied cases, irreversible fouling did not arise on the membranes. This was not the case as soon as olive mill wastewater (OMW) was treated: due to the high concentration of pollutants, this wastewater fouls the membrane very quickly, within days of operation, if no pretreatment is carried out beforehand [14].

Proper and optimal designed pretreatment processes on the given feedstock must be developed in order to maximize productivity and minimize fouling: this research objective will be referred to from now on as the concept of pretreatment tailoring of membrane processes.

In this work, previously measured critical and threshold flux data by the Authors will be analyzed again in order to check the possibility to merge both concepts under one common hat, which is the boundary flux. The complete and available dataset of the authors on studies performed on OWW and OMW exiting the two-phase and three-phase olive oil extraction processes is sufficient to allow reaching conclusions which should be generally valid for other systems too.

At first, both critical and threshold flux equations will be discussed. Successively, common and different characteristics of the two concepts will be sorted out. Finally, the application of the new concept, that is, the boundary flux, will be presented and validated.

## 2. The Critical and Threshold Flux Concept and Equations

Concerning the critical flux *J*_*c*_, hereafter used in terms of critical flux for irreversibility, the following fitting equations apply [5]:

(1) $$\begin{array}{c} \frac{dm}{dt}=0;\;J_{p}\left(t\right)\leq J_{c}, \end{array}$$

(2) $$\begin{array}{c} \frac{dm}{dt}=B\left(J_{p}\left(t\right)-J_{c}\right);\;J_{p}\left(t\right)>J_{c}, \end{array}$$

where *m* is the permeability of the membrane, *B* is a fitting parameter, and *J*_*p*_(*t*) is the permeate flux at time *t*.

Concerning the threshold flux *J*th, the proposed equations by Field et al. are as follows [7]:

(3) $$\begin{array}{c} \frac{dm}{dt}=a;\;J_{p}\left(t\right)\leq J\text{th}, \end{array}$$

(4) $$\begin{array}{c} \frac{dm}{dt}=a+b\left(J_{p}\left(t\right)-J\text{th}\right);\;J_{p}\left(t\right)>J\text{th}, \end{array}$$

where *a*, *b* are both fitting parameters.

It is interesting to notice that the threshold flux equations are similar to the critical flux equations and differ only by the presence of the “*a*” parameter. In fact, if the case of *a* = 0 is admitted, (3) and (4) may reduce to (1) and (2), respectively.

The parameter “*a*” value measures below threshold flux conditions the constant permeability loss rate of the membrane in time. If this value is equal to zero, no permeability will be lost in time and therefore no fouling is triggered. This is valid only below critical flux conditions, and therefore (3) includes (1) if *a* = [0, *∞*).

Above critical and threshold flux conditions, fouling behaves in similar way by exponential permeability loss rates in time. Again, if *a* = [0, *∞*), (4) fits (2). The only difference between these systems is that in critical flux characterized systems fouling is not affected by the continuous presence of a constant fouling permeability loss rate as in threshold flux characterized systems. Beside this theoretical difference of the two systems, the authors want to point out that this aspect is of limited practical importance, since the exponential part of (2) and (4) will quickly overwhelm the linear contribution of the parameter “*a*” in (4).

Summarizing, both critical and threshold fluxes divide the operation of membranes into two regions: a lower one, where no or a small constant amount of fouling may form, and a higher one, where fouling builds up very quickly. By introducing a new flux, that is, the boundary flux *J*_*b*_, the previous equations may be written as

(5) $$\begin{array}{c} \frac{dm}{dt}=-\alpha ;\;J_{p}\left(t\right)\leq J_{b}, \end{array}$$

(6) $$\begin{array}{c} \frac{dm}{dt}=-\alpha +\beta \left(J_{p}\left(t\right)-J\text{th}\right);\;J_{p}\left(t\right)>J_{b}, \end{array}$$

where

- *α*, expressed in [L h^−2^ m^−2^ bar^−1^], represents the constant permeability reduction rate suffered by the system and will be hereafter called the subboundary fouling rate index.
- *β*, expressed in [h^−1^ m^−2^ bar^−1^], represents the fouling behavior in the exponential fouling regime of the system and will be hereafter called superboundary fouling index.

The method used to measure the boundary flux is similar to the ones used to measure critical flux values but needs a different approach in order to determine the value of *α* at first and the value of *β* successively. Beside experimental data, the extended method requires the use of (5) and (6) to separate the two operating regimes.

## 3. The Boundary Flux Concept

The introduction of the new boundary flux concept does not extend by addition of new theory or knowledge the critical and threshold flux concepts. On the other hand, it tries to simplify the use of these concepts in future works. Referring to one single concept will reduce sensibly the incorrect use of both the critical and threshold flux concepts.

The authors suggest the use of the pressure stepping method, extended from the one used by Espinasse et al. in previous works [16]. This method was found to be reliable and relatively quick. Basically, the method consists of applying different pressure values by periodic changes after a specific time interval, and following a predetermined scheme, characterized by a specific difference of pressure equal to ΔTMP. At first, pressure value is increased by 2ΔTMP; after this, reduced by ΔTMP. As an example, in case of ΔTMP equal to 1 bar, the series of applied pressure values would be: 2 bar, 3 bar, 2 bar, 4 bar, 3 bar, 5 bar, 4 bar, 6 bar and so on. Every time the same pressure value was set for the second time in one series, a reproducibility check of the permeate flux is performed and differences must be notified. Moreover, the method requires the integration of (5) in time, as follows:

(7) $$\begin{array}{c} J_{b}\left(\text{TMP},t1\right)-J_{b}\left(\text{TMP},t2\right)=-\Delta J_{b}^{*}=-\alpha \text{TMP}\left(t2-t1\right), \end{array}$$

which is valid in case the same TMP value is used at *t*1 and *t*2. It is possible to use different TMP values between *t*1 and *t*2 without invalidating (7): as long as the adopted TMP values remain below the boundary one, no effect on changes of the permeability loss rate should be observed. Equation (7) is again valid for both critical and threshold fluxes, and as long this equation holds, boundary conditions are met. As a consequence, the definition of boundary flux is as follows.

The lowest pressure value at which the difference between the measured −Δ*J*_*b*_ and the evaluated value from (7) −Δ*J*_b_* in the same period of time become positive is the boundary pressure TMPb, and the boundary flux value is determined by taking into account the permeate flux value at the beginning of the correspondent pressure cycle.

The boundary flux values are sensibly influenced by those parameters affecting the critical and threshold flux, hereafter listed as follows:

- hydrodynamics,
- temperature,
- membrane properties,
- feedstock characteristics.

On the first three points much has been reported in the literature and the effects are nowadays well known [7]. Moreover, it is relatively simple to maintain these parameters constant during membrane operation once fixed.

This is not the case of the last point, which merits some more attention. Firstly this parameter is sensibly affected by pretreatment processes. A good design of the pretreatment processes (called pretreatment tailoring) beforehand the membrane section appears to increase *J*_*b*_ accordingly. A correct pretreatment tailored for membranes is a difficult task, since the modification that occurs on the feed stream must be valuable for all successive membranes.

Some researchers use microfiltration (“MF”) as pretreatment for the successive membrane steps [17, 18]. Although this approach saves UF and NF from sensible fouling, the MF sustains a heavy duty. Fouling problems on membranes results to be shifted on only one separation step, and makes the overall process difficult. Using ceramic or tubular modules makes the cleaning of membranes easier but still does not solve the problem due to high cleaning procedure costs [19]. The best strategy is therefore to distribute fouling among all involved membrane steps equally. Pretreatment processes should be therefore of physical or chemical nature.

As an example, the application of fungi for organic matter reduction works well for UF, but due to the production of enzymes of a size near the NF pore size, on this latter membrane, the threshold flux values sensibly drop [15]. As a consequence this pretreatment does not fully qualify for the process. Better results may be obtained by adopting microalgae, but this process is still under investigation [20]. Moreover, membrane bioreactors has been investigated, with promising results, but characterized by insufficient productivity [21, 22]. The same problem affects membrane distillation based processes [23]. Flocculation by heavy metal salts efficiently increases threshold flux values on all membranes, but in case of OWW treatment, heavy metal ions still remain and are measured in the reverse osmosis permeate: again, the pretreatment does not qualify for legislative issues for wastewater recovery [24]. This is not a general rule: in case of treating by flocculation tomato vegetation wastewater or marine sediments, these problems were not encountered [25, 26]. Flocculation was therefore developed by substituting the flocculent with nitric acid. The obtained benefit in threshold flux value increases was reduced, but the problem of heavy metal ions in the permeate was overcome [27]. Photocatalysis appears to be less efficient than flocculation but increases threshold flux values of all membranes and allows producing a purified water stream compatible to the sewer system due to organic matter reduction and organic chain cutting [28]. Photocatalysis was studied both as Fenton process or by using titania nanocatalyst [29–31]. Problems here are the high operating costs of the reactants, making the process economically unfeasible. The solution here was to use magnetic core nanocatalyst, which can be completely recovered back by a magnetic trap and reused in successive batches [32].

The optimized application of different pretreatment processes inhibits differently the fouling processes triggering over the membrane surface, and fouling indexes may enter a technically sustainable range [33]. By using OWW, only threshold flux and no critical flux values were found and measured. The data can be used for process optimization purposes [34, 35]. This permits maintaining membranes efficient for very long period of time [36].

During all these works it was observed that fouling is mainly given by both dissolved and suspended solutes, and since tracking these parameters is normally a difficult task, a key parameter must be chosen to define a fingerprint of the feedstock, useful to fit to *J*_*b*_ values.

In the previous works the fitting curve chosen by the authors was as follows [36]:

(8) $$\begin{array}{c} J_{b}\left(\text{KP}\right)=J_{b}\left({\text{KP}}_{\text{ref}}\right)-B\ln\left(\text{KP}\;{{\text{KP}}_{\text{ref}}}^{-1}\right), \end{array}$$

where KP is the value of the chosen key parameter and KP_ref_ is the value of the experimentally determined reference data point. Although this fitting curve satisfied the process optimization needs successfully, it has the limit to be determined by statistical analysis of the available experimental data. The main concern of this fitting curve is the limit value for KP tending to zero, equal to

(9) $$\begin{array}{c} \lim\left[\text{KP}⟶0\right]J_{b}\left(\text{KP}\right)=\infty , \end{array}$$

which has no direct physical justification. In fact, this value should be limited and equal to the observed pure water permeability value. Moreover, the logarithmic function does never reach zero flux conditions even at very high KP values, and this is in contrast to the literature.

At this point the authors want to propose another fitting curve based on the general relationship between permeate flux *J*_*p*_ and TMP; that is,

(10) $$\begin{array}{c} J_{p}\left(\text{KP},t\right)=m\left(\text{KP},t\right)\text{TMP}\left(\text{KP}\right), \end{array}$$

where *m*(KP, *t*) is the permeability and TMP(KP) is the transmembrane pressure, as a function of the chosen key parameter at a given time *t*. Considering the key parameter representative of the concentration beside particle size of the solute in the feedstock solution, both *m* and TMP can be approximated by a linear function:

(11) $$\begin{array}{c} m\left(\text{KP},t\right)=m0\left(t\right)-m1\text{KP},\\ \text{TMP}\left(\text{KP}\right)=P-\text{KP}RT=P-p1\text{KP}, \end{array}$$

where *P* is the applied operating pressure, *R* is a constant, *T* is the temperature, and *p*1 and *m*1 are fitting parameters. The pure water permeability, that is, *m*0(*t*), is a function of time since it depends on the amount of irreversible fouling formed over the membrane. Substituting at boundary conditions (5) (with *m*0 being the pure water permeability at *t* = 0 and *P*_*b*_ the boundary operating pressure), (11) in (10), the following relationship is obtained:

(12) $$\begin{array}{c} J_{b}\left(\text{KP},t\right)=\left[m0-m1\text{KP}\right]\left[-\alpha t\right]\left[P_{b}-p1\text{KP}\right], \end{array}$$

and finally

(13) Jb(KP,t)=m0Pb−αtPb−[m0p1−αp1t+m1Pb]KP+m1p1KP2.

The obtained relationship is a second order polynomial equation, valid in the physical range of KP = [0, +*∞*) and qualifies as a better fitting equation because the following reasons.

- The limit has now a physical meaningful limit at pure water conditions; that is, lim⁡[KP → 0]*J*_*b*_(KP, *t*) = *m*0(*t*).
- The fitting curve is always convex, since (*m*1*p*1) > 0, and has always two roots. The lowest one will be called hereafter KP*, which represents the upper limit of solute concentration in the feed solution to trigger almost instantaneously zero flux conditions.
- For [*t* → *∞*], the minimum point tends to −*∞*; in other words, the relevant *J*_*b*_(KP) values are dependant of time and reduces. By looking at the plot of the *J*_*b*_(KP) curve, this latter one tends to shift downwards as a function of time. There is a time point t* for a given KP where zero flux conditions are immediately met and thus the module is completely dead. In fact, the reason for this is the irreversible fouling build up and/or aging of the membrane.
- Generally, *J*_*b*_ is a nonconstant value; it depends directly on time, as a function of *α*. Theoretically, the value of *J*_*b*_ should be continuously measured. This is not possible in practice, and as a consequence, the use of raible boundary flux models as a function of time and KP appears necessary.
- On the other hand, the operating boundary pressure *P*_*b*_, and as a consequence the value of TMPb if KP is constant, is not a function of time. This particular behavior was previously observed in some works by the authors [37, 38].
- The importance of the findings reported at point (d) and point (e) relays in the choice of the control system of the process. If the control strategy consists in maintaining the operating pressure constant, the permeate flux will follow the relevant *J*_*b*_(*t*) profile as long as the KP value does not change. Generally, membrane processes are controlled by controlling the permeate flux rate in order to assure the project value of productivity: in this case, the use of boundary flux models is mandatory.
- Although at high KP values the fitting curve tends to positive *J*_*b*_ values, this part of the curve has no physical meaning; therefore, the validity of the fitting curve must be restricted to KP values in the range [0, KP*].

**Figure 1:**
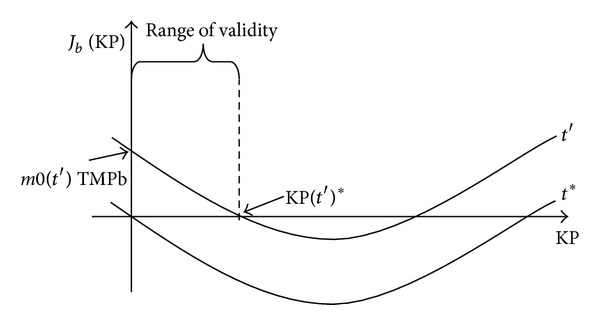
Plot of (13) at fixed time points *t*′ and t*.

Figure 1 summarizes all the characteristic points of the new fitting equation, that is (13).

## 4. Validation of the Boundary Flux Concept by Previously Published Experimental Data

Hereafter, the use of the new boundary flux concept will be discussed by the analysis of previously published experimental data by the Authors. The dataset consists of critical and threshold flux measurements performed during the treatment of OWW and OMW, respectively. OWW was pretreated by coagulation with aluminum sulphate before the membrane section. The pretreatment for both OMW streams was the same and consists in an optimized coagulation by nitric acid addition and a photocatalysis process by titania nanoparticles, described in detail elsewhere [39, 40]. The used pilot plant and membrane modules were the same in all experiments (see Table 1).

In case of OWW, electric conductivity (“EC”) appears to be sufficient; alternatively, the COD values can be taken into account. In case of OMW, this simplification appears to be not sufficient, and the best key parameter appears to be a combination of the COD value of the feed solution and the contained amount of interfering suspended particles with the membrane pores, expressed as percentage, and hereafter called “*μ*p.” The units are [% mg L^−1^].

**Figure 2:**
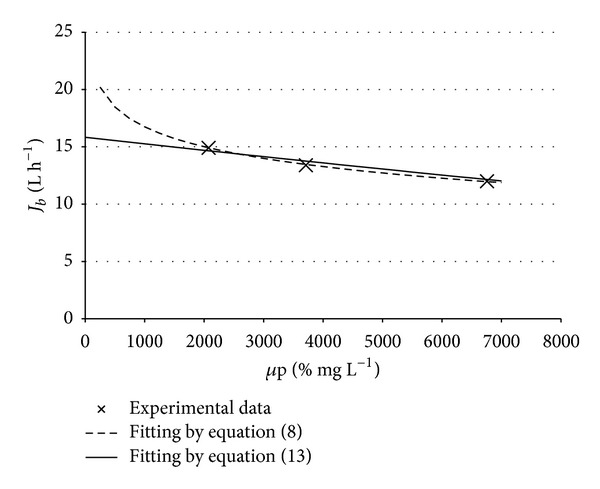
Comparison of the old fitting curve by (8) and the new fitting curve by (13).

To permit validation of the new fitting curve by (13) in Table 2 the relevant data and in Figure 2 both fitting curves from (8) (in this case an optimal fitting value of *B* = 2.5 was found) and (13) are reported by using a previous experimental dataset taken during the treatment of OMW3 by NF. The values for KP* and t* were evaluated and reported too.

The obtained results are satisfactory, since many times the authors reported that without any pretreatment, that is, at *μ*p values higher than 44000% mg L^−1^, almost instantaneous zero flux conditions are met. Moreover, the value of t* is confirmed by the author's personal experience: the process can hold on approximately 96 h before immeasurable permeate flow rates are observed and washing is required.

In Table 3, the different feedstock characteristics (raw, after pretreatment if any before UF, before NF) used during the different experimental runs are reported, respectively.

**Figure 3:**
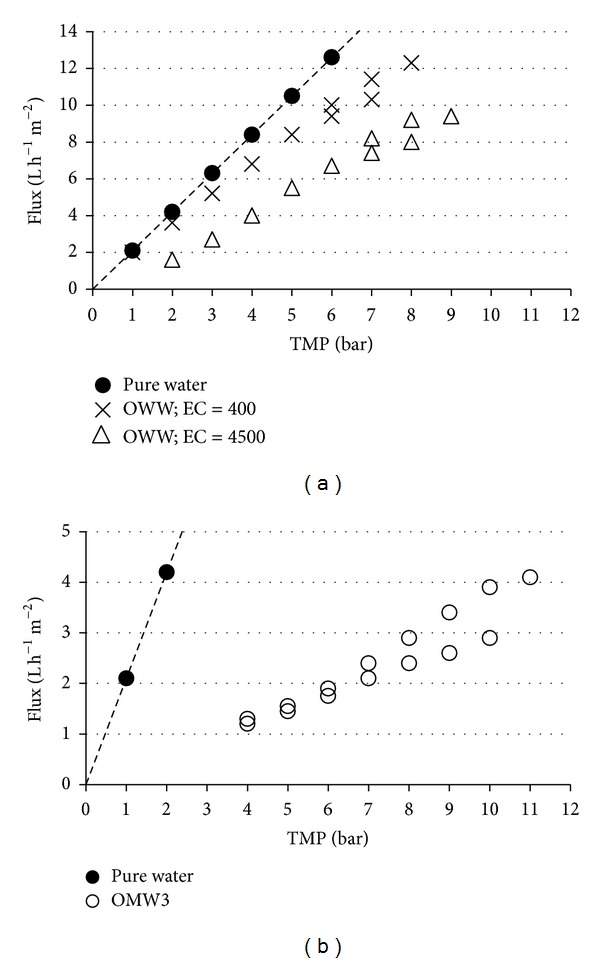
Comparison of the boundary flux measurement patterns found on NF for OWW (a) and OMW3 (b).

Although OWW and OMW exit from the same process line type, that is, olive oil production, they have many differences. OWW is a slightly polluted wastewater stream, with a small amount of dissolved organic matter content and a high amount of suspended solids characterized by a very big particle size; OMW is a heavily polluted wastewater stream, with a high amount of dissolved organic matter content and a very high amount of suspended solids characterized by a very small particle size, similar to the membrane's pore size. The different composition of the feedstock gives rise to a different fouling potential and therefore different *J*_*b*_ measurement patterns. For sake of example, Figure 3 reports boundary flux measurements performed on NF by using OWW Figure 3(a) and OMW3 Figure 3(b).

The final results of the measured boundary fluxes and all relevant parameter values are summarized for UF and NF in Tables 4 and 5, respectively.

The parameter mw%, that is, the percentage loss of the pure water permeability in 1 hour of continuous operation, includes in theory membrane aging and the small amount of irreversible fouling triggering on the membrane, even below boundary flux conditions. Membrane aging is not measurable in this period of time and should be neglected. This data is sufficient to properly design the membrane plant following the procedure described elsewhere [27, 33].

In case of OWW, at low operating conditions, fouling is not observed. The value of *α* is equal to zero for both UF and NF, thus stating initial beyond critical flux conditions on both membranes. As a function of the key parameter EC, that is, the measured electoconductivity of the feedstock, different *J*_*b*_ values are measured.

This is not the case of OMW, where the value of *α* appears different from zero even at low TMP values, for UF and NF. Therefore, the presence of a threshold flux is suspected. The analysis shows for different COD values different *α* and *J*_*b*_ values. In particular, OMW2 appears to raise less severe fouling on both UF and NF membranes, exhibiting always both lower *α* and higher *J*_*b*_ values.

## 5. Conclusions

This paper wants to set a milestone in the use of combined critical and threshold flux concepts to membrane process plant design. The introduction of the boundary flux, which avoids incorrect use of terms and definition and merging both concepts into one fits perfectly this need. Moreover, the boundary flux shares the same characteristics and is affected by the same influence of variables affecting critical and threshold flux. In particular, the relationship between boundary flux and the pollutant type and concentration in the feed solution was furthermore exploited and a new fitting curve was proposed. The suggested approaches were finally validated by both experimental data of previous works published by the authors on the treatment of olive wash and olive mill wastewater streams.

## Figures and Tables

**Table 1:** Experimental setup for UF and NF, valid for all feedstock.

	UF	NF
Feed flow rate [L h^−1^]	600	600
Temperature [°C]	20 ± 1	20 ± 1
Membrane ID	Desal Osmonics GM2540	Desal Osmonics DK2540
Module type	Spiral wounded	Spiral wounded
Membrane area [m^−2^]	2.51	2.51
Average pore size [nm]	2.0	0.5

**Table 2:** Input data for (13) evaluation.

	Fitting by new (13)
*m*0 [L h^−1^ m^−2^ bar^−1^]	2.2
*m*1 [—]	5 10^−5^
*p*1 [bar L %^−1^ mg^−1^]	1 10^−4^
*α* [L h^−2^ m^−2^ bar^−1^]	0.02
*P*_*b*_ [bar]	7.2
KP*[% mg L^−1^]	44000
t*[h]	110

**Table 3:** Feedstock characteristics.

		EC [*μ*S cm^−1^]	COD [mg L^−1^]	mp [—]	pH [—]
OWW	RAW	850	755	560	5.3
	UF	943	715	402	6.8
	NF	610	357	198	7.5

OMW2	RAW	1910	16600	N/a	4.9
	UF	1800	11000	9625	2.9
	NF	745	5700	4132	3.1

OMW3	RAW	6370	50100	42084	5.5
	UF	7520	25100	15060	3.0
	NF	3698	5460	2075	3.0

**Table 4:** UF boundary flux values for OWW, OMW2, and OMW3.

	OWW	OMW2	OMW3
TMPb [bar]	2.2	10	4.0
*J*_*b*_ [L h^−1^]	6.2	10.0	5.1
*α* [L h^−2^ m^−2^ bar^−1^]	0.0000	0.0110	0.0553
mw% [% h^−1^]	0.0000	N/a	0.016
Boundary flux type	Critical flux	Threshold flux	Threshold flux

**Table 5:** NF boundary flux values for OWW, OMW2, and OMW3.

	OWW	OMW2	OMW3
TMPb [bar]	6.0	9	8.0
*J*_*b*_ [L h^−1^]	4.2	14.3	7.7
*α* [L h^−2^ m^−2^ bar^−1^]	0.0000	0.0050	0.0191
mw% [% h^−1^]	0.0000	N/a	0.009
Boundary flux type	Critical flux	Threshold flux	Threshold flux
